# Improving Transungual Permeation Study Design by Increased Bovine Hoof Membrane Thickness and Subsequent Infection

**DOI:** 10.3390/pharmaceutics13122098

**Published:** 2021-12-06

**Authors:** Sebastian Kappes, Thilo Faber, Lotta Nelleßen, Tanju Yesilkaya, Udo Bock, Alf Lamprecht

**Affiliations:** 1 Department of Pharmaceutics, Institute of Pharmacy, University of Bonn, Gerhard-Domagk-Str. 3, 53121 Bonn, Germany; kappes.sebastian@uni-bonn.de (S.K.); thilo.faber@uni-bonn.de (T.F.); s6annell@uni-bonn.de (L.N.); 2 Bayer Vital GmbH, 51373 Leverkusen, Germany; tanju.yesilkaya@bayer.com; 3 Bock Project Management, 54456 Tawern, Germany; udo.bock@bock-pm.com; 4 PEPITE EA4267, University of Burgundy/Franche-Comté, 25000 Besançon, France

**Keywords:** ungual permeation model, onychomycosis, bovine hoof, bifonazole, scanning electron microscopy

## Abstract

Ungual formulations are regularly tested using human nails or animal surrogates in Franz diffusion cell experiments. Membranes sometimes less than 100 µm thick are used, disregarding the higher physiological thickness of human nails and possible fungal infection. In this study, bovine hoof membranes, healthy or infected with *Trichophyton rubrum*, underwent different imaging techniques highlighting that continuous pores traversed the entire membrane and infection resulted in fungal growth, both superficial, as well as in the membrane’s matrix. These membrane characteristics resulted in substantial differences in the permeation of the antifungal model substance bifonazole, depending on the dosage forms. Increasing the thickness of healthy membranes from 100 µm to 400 µm disproportionally reduced the permeated amount of bifonazole from the liquid and semisolid forms and allowed for a more pronounced assessment of the effects by excipients, such as urea as the permeation enhancer. Similarly, an infection of 400-µm membranes drastically increased the permeated amount. Therefore, the thickness and infection statuses of the membranes in the permeation experiments were essential for a differential readout, and standardized formulation-dependent experimental setups would be highly beneficial.

## 1. Introduction

Almost five percent of the North American and European population is affected by fungal nail infection, mainly occurring on the toenails [1]. Such infections are mostly caused by dermatophytes and, to a lesser extent, by yeasts or molds. The dermatophyte *Trichophyton rubrum* (*T. rubrum*) alone accounts for about 90% of cases overall [2]. Common symptoms, including discoloration, brittleness, and deformation of the nail plate, can affect one’s psychological well-being and, in severe cases, even lead to social isolation. If left untreated, such infections may spread to live tissue, promoting superinfections with additional microbial pathogens, ultimately causing pain and possibly impacting patients’ mobility and general quality of life [3]. Despite the availability of highly effective antifungal agents, treatment is often insufficient, with high recurrence rates [4], as orally available actives carry the risk of severe side effects, and topical treatment usually requires monthlong treatment. This is due to the excellent barrier function of the nail, which consists of fused, flattened keratinocytes. The lipid content in the nail is low compared to the stratum corneum, and the resulting matrix can generally be described as hydrophilic [5,6]. Fungi growing inside and below the nail can therefore hardly be reached by active agents, as their typically highly lipophilic nature limits their partitioning into and diffusion through this matrix.

With cases of onychomycosis increasing and problematic drug-resistant strains of pathogens emerging [7,8], the development of formulations that surpass the limits imposed by the nail barrier has become increasingly important. Hence, adequate test systems to reliably assess the delivery of actives out of these formulations are needed. Typically, permeation studies are performed using Franz diffusion cells (FDC) equipped with different membranes. While human nails would provide the most realistic results, human cadaver nails require specialized diffusion cell geometry because of their curvature and are limited in availability. Besides the infectious risk, the variability of nail thicknesses and surface areas may lead to the high variability of study outcomes. Alternatively, nail clippings still do not eliminate an impractical curvature and provide only a small diffusion area, constraining their regular use in FDC setups. Synthetic membranes may enable the assessment of intrinsic release properties of formulations. However, they lack the physiological properties of a keratinous matrix, disregarding, for instance, the protein binding of substances, and are only a few micrometers thin. This disregard for the special density and properties of the keratinous matrix would lead to a high overestimation of permeations in vivo.

Therefore, bovine hoof sheets (BHS) were established as an in vitro surrogate for human nails in transungual permeation studies, as they are composed of α-keratin with a high sulfur content, comparable to human nails [9]. Following the established protocols for production resulted in membranes with reproduceable thicknesses and homologous morphology, possibly reducing the variance of in vitro permeation results [10]. Usually, 100–200-µm-thick BHS are used in permeation studies. Since, however, human nails are typically between 250 µm and 600 µm thick [11], such a thin BHS might also lead to the overestimation of antifungal drug permeation, due to a lack of barrier thickness and integrity. In addition, these models disregard the pathological state of nail infections, since they are mechanically, not sufficiently, stable after infection, which presumably impacts the permeation behaviors of actives.

In this work, it was investigated whether increasing the healthy BHS dimensions from 100 µm to a more physiologically relevant thickness of 400 µm and the subsequent infection with *T. rubrum* would be beneficial in providing ameliorated mechanical properties. In parallel, we elucidated to what extent the flux from different ungual dosage forms is impacted by such a difference in BHS thickness. For this purpose, bifonazole was chosen as an antifungal model substance and formulated as an ethanolic solution, nail patch, and nail lacquer with equal drug load, which were tested for their respective permeation behaviors. Two commercial products: Canesten^®^ Extra Salbe and Canesten^®^ Extra Creme served as the semisolid references. For interpretation of the results, extensive characterization of the BHS was conducted with different imaging techniques.

## 2. Materials and Methods

### 2.1. Materials

Bifonazole (BFZ) was purchased from TCI Deutschland GmbH (Eschborn, Hesse, Germany). Canesten^®^ Extra Creme (CRE) and Canesten^®^ Extra Salbe with (SAL) and without urea (SAL *w*/*o*) were kindly donated by Bayer Vital GmbH (Leverkusen, Germany). DuroTAK 2054 (DT) was a gift from Henkel AG & Co. KGaA (Düsseldorf, Germany), and Eudragit RL PO (RLPO) was donated by Evonik Industries (Essen, Germany). A spectra/Por^®^6, molecular weight cutoff 50-kDa dialysis membrane was purchased from Carl Roth (Karlsruhe, Germany). A ScotchPak^TM^ 9733 backing liner was kindly donated by 3M (St. Paul, MN, USA). Ethanol absolute 99.8% was purchased from VWR International GmbH (Darmstadt, Germany). Triacetin, sodium phosphate dibasic dihydrate, and sodium chloride were purchased from Sigma Aldrich (Steinheim, Baden-Württemberg, Germany). Potassium dihydrogen phosphate was purchased from Carl Roth (Karlsruhe, Germany). Potassium chloride was purchased from Merck (Darmstadt, Germany). The chemicals used for the dosage form or buffer preparation were of analytical grade or higher.

### 2.2. Preparation of Solution (SOL) 

One percent (*w/w*) BFZ was dissolved in absolute ethanol at room temperature under stirring until a clear solution was obtained.

### 2.3. Preparation of Lacquer (LAC)

Twenty percent (*w/w*) RLPO and 1% (*w/w*) BFZ referring to the dry film mass were completely dissolved in ethanol under stirring, and 5% (*w*/*w*) triacetin was added subsequently analogous to Yang et al. [12]. A solid-state analysis was conducted using escalated drug loads of 10 and 20% (*w*/*w*) to enhance the sensitivity of the methods to recrystallization in the matrix.

### 2.4. Preparation of Patch (PAT)

One percent (*w*/*w*) BFZ related to the solid content of an adhesive in the solution was added to the matrix and dissolved under stirring. The solution was then cast onto backing liner with a wet film applicator (Multicator 411, Erichsen, Hemer, Germany) with a wet film thickness of 1000 µm. The patches were allowed to rest at an ambient temperature for 15 min and then dried at a temperature of 65 °C for 30 min. Before application, the patches were cut into rectangular pieces of 2 cm^2^. The patch matrices with enhanced drug loads of 10 and 20% (*w*/*w*) were prepared to increase the sensitivity of the methods and were subsequently assessed for crystallinity.

### 2.5. Polarized Light Microscopy (PLM)

PLM images were taken with a Leica DM 2700M (Leica microsystems, Wetzlar, Germany) equipped with a MicroPublisher 5.0 RTV camera (Teledyne Photometrics, Tucson AZ, USA). Images were captured using Q-Capture Pro 7 software version 7.0.5. Samples were prepared by casting the respective matrix directly onto microscope slides, followed by the same drying procedure employed in preparation of the final dosage forms. 

### 2.6. Differential Scanning Calorimetry (DSC)

DSC measurements were conducted using a Mettler-Toledo DSC 2 (Gießen, Germany) that was equipped with a nitrogen cooling system using nitrogen as the purge gas (30 mL/min). Samples were weighed on aluminum pans at 5–15 mg and closed with a pierced lid. The glass transition temperature and melting point for neat BFZ were determined using a heat–cool–heat cycle (25 °C to 170 °C to −50 °C to 170 °C) with a heat rate of 10 K/min. The glass transition temperatures and melting points for the polymer films and patch matrices were investigated using TOPEM mode with a heat rate of 2 K/min from −70 °C to 170 °C with a pulse height of 1 K.

### 2.7. X-ray Diffraction (XRD)

XRD was performed in transmission mode on X’Pert MRD Pro by PANalytical (Almelo, The Netherlands) equipped with an X’Celerator detector. Nickel-filtered CuKα1 radiation was generated at 45 kV and 40 mA. Scanning was performed in a range from 17° to 20° 2Θ containing the most prominent reflection peaks, with a step size of 0.017°.

### 2.8. X-ray Microtomography (µCT)

µCT scans of 400 µm BHS were performed using Bruker SKYSCAN 1272 (Kontich, Belgium) equipped with a Hamamatsu L11871-20 x-ray source and a XIMEA xiRAY16 camera with a pixel size of 7.4 µm. The membranes were first submerged in 2.5% (*w*/*v*) glutaraldehyde solution for fixation and subsequently put in 2% (*w*/*v*) OsO_4_ solution to increase the contrast. The scanning parameters were set to 60-kV source voltage, the current to 166 µA, a 0.25-mm Al filter, an image rotation of 188.4° with a step size 0.4°, a 1900-ms exposure time, and about 1 h 8 min of scan time. Initial image reconstruction was conducted with NRecon software version 1.7.1.0 (Kontich, Belgium). Volume rendering and stack analysis of the figures shown were performed using free ImageJ software version 1.53c (https://imagej.nih.gov/ij/ accessed on 11 December 2020).

### 2.9. Preparation of Bovine Hoof Sheets

Cattle hooves for membrane preparation were procured from a local abattoir on the day of slaughter for human consumption. As these were a byproduct in proper food production and animals were not bred, kept, or sacrificed for scientific purposes but for food production only, ethics committee approval was not required. The membranes were prepared analogous to the method described by Mertin and Lippold [13]. In brief, hooves were cut into 2 cm × 2 cm squares, submerged in distilled water for 3 d, and subsequently cut with a rotational microtome to 100-µm and 400-µm-thick membranes.

### 2.10. Scanning Electron Microscopy (SEM) and Focused Ion Beam Scanning Electron Microscopy (FIB-SEM)

Infected and healthy sheets were glued to aluminum stubs with silver conductive paint and sputter-coated with platinum (Q150TS Quorum Technologies, Lewes, UK).

Electron microscopy and focused ion beam milling were performed using a Helios G4 CX Dualbeam microscope (Thermo Fisher Scientific, Eindhoven, The Netherlands). Secondary electron images were taken using a 2–10 kV accelerating voltage at 4 mm working distance. Rough Fib milling was performed on stained membranes using 30-kV accelerating voltage and a 9.3-nA current. The fine polishing of the revealed cross-section was done at 30 kV and a 43-pA current.

### 2.11. Infection of Bovine Hoof Sheets

BHS were infected with *T. rubrum* over a period of 9 d. Infection was performed according to a slightly modified procedure by Lusiana et al. [14]. Inoculation of hoof sheets was started by 30-min maceration in a physiological saline solution. Sabouraud–Agar plates (Becton Dickinson GmbH, Heidelberg, Germany) with gentamicin and chloramphenicol were struck with a cotton swab containing *T. rubrum* in a physiological saline solution in one direction, applying a zig-zag pattern. Agar plates were dried for 10 min. Subsequently, BHS were placed on top and their surfaces moistened with the same swab. BHS were incubated for 9 days at 30 °C ± 2 °C. After colonization, the BHS were disinfected by 3 h of treatment in 70% (*v*/*v*) ethanol and subsequent UV exposure for 1 h. Membranes with similar colors, corresponding to the level of infection without visible damage such as cracks, were selected for further experiments.

### 2.12. Permeation across Synthetic Membranes

Release from the formulations was assessed using vertical Franz diffusion cells (FDC) with an acceptor volume of 8 mL and diffusion area of 1 cm^2^. A regenerated cellulose membrane with a molecular weight cutoff of 50 kDa was mounted between the donor and acceptor compartments. To achieve sink conditions throughout the experiment, the receiver consisted of 42% (*v*/*v*) ethanol in phosphate-buffered saline, pH 7.4 [15] that was stirred at 200 rpm, and the FDC were maintained at 32 °C. A dose of 200 mg in the cases of SAL, CRE and SOL was applied to the membrane. Three hundred and fifty microliters of LAC were applied to the donor as the solution and allowed to dry overnight before filling the receiver to start the experiment. PAT were applied directly onto the the membrane before insertion into FDC. Samples of 0.5 mL were taken hourly up to 6 h and after 24 h and analyzed by HPLC-DAD. The removed volume was immediately replaced with fresh receiver solution.

### 2.13. Permeation across Bovine Hoof Sheets

Permeation studies in vitro were carried out over 6 days in vertical FDC with an acceptor volume of 5.5 mL and a diffusion area of 0.785 cm^2^. The other conditions were kept in accordance to experiments with synthetic membranes. BHS were hydrated in the receiver for 24 h prior to insertion into the FDC. The applied dose for each dosage form was analogous to the synthetic membrane experiments, and the donor was replaced daily for SAL, CRE and SOL with cotton swabs to account for the higher depletion of BFZ in immediate release formulations. Due to the low adherence of SAL to swabs, the residual donor was further removed by the careful use of a plastic spatula. Samples of 0.5 mL were taken daily after 24 h and analyzed by HPLC-DAD. A removed volume was immediately replaced with a fresh receiver solution.

### 2.14. High-Performance Liquid Chromatography (HPLC) Analysis

An analysis was conducted on an Agilent 1100 system coupled with an Agilent G1316A detector using a C18 reverse-phase column (LiChrospher^®^ 100 RP 18 EC–5µ; 125-mm length, 4.6-mm inner diameter, and 5-µm particle size). The eluent consisted of acetonitrile and 20-mM sodium phosphate buffer, pH 7.4 (70/30, % *v*/*v*) set to a flow of 1 mL/min. The column temperature was maintained at 25 °C, and the autosampler was left at room temperature. The injection volume was 40 µL, and the detector was set to 256 nm. The limit of quantification was 100 ng/mL. Its specificity was ensured by injection of a BFZ standard from methanol and from a FDC receiver medium, aliquots of dosage forms, pure receiver medium, and receiver medium previously incubated with BHS. No peaks interfered with the BFZ peak. The following parameters were evaluated: slope = 169.0; y-intercept = −1.550; R^2^ = 0.9993; linear range = 0.1–11 µg/mL; retention time = 4.5 min; repeatability = 0.4%; interday variations = 2.1%; and accuracy = 104 ± 1.4%, 94.7 ± 1.5%, and 97.5 ± 0.5% for concentrations of 0.2, 1.5, and 4 µg/mL, respectively.

### 2.15. Statistical Evaluation

All experiments were conducted in triplicate at least. The flux was calculated using at least three terminal linear data points. Data were presented as the mean ± standard deviation. A statistical analysis for significance was conducted using GraphPad Prism 8 software Welch’s *t*-test. The results were recognized as statistically significant if *p* < 0.05 and marked with an asterisk. The results were considered very significant if *p* < 0.01 and extremely significant if *p* < 0.001 and marked with two or three asterisks, respectively.

## 3. Results

### 3.1. Image Characterization of Bovine Hoof Membranes

**Figure 1:**
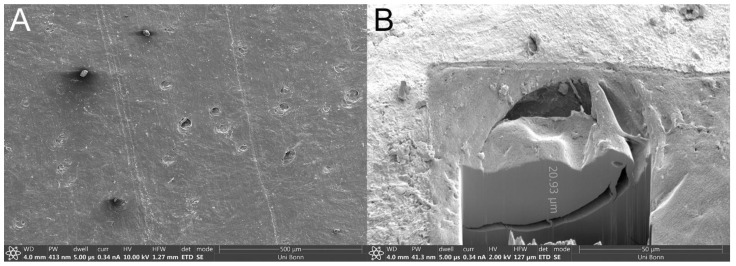
Scanning electron microscopy images of a bovine hoof sheet surface: (**A**) porous structures spread over the entire surface and (**B**) exemplarily focused ion beam milling of a pore reveals continuation into the membrane matrix.

**Figure 2:**
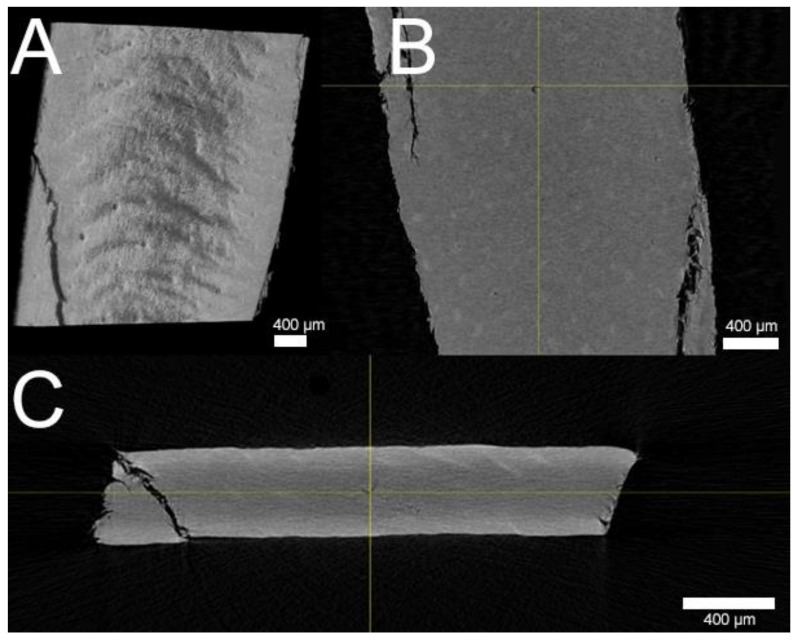
Volume rendering of x-ray microtomography bovine hoof sheet (BHS) images. (**A**) Surface of BHS in the Y-direction with various porous cavities visible. (**B**) Focused hollow pore (yellow cross) inside the BHS matrix in the Y-direction and (**C**) Z-direction, revealing continuation of the pore as a dark crack from the top left to bottom right; each scale bar = 400 µm.

**Figure 3:**
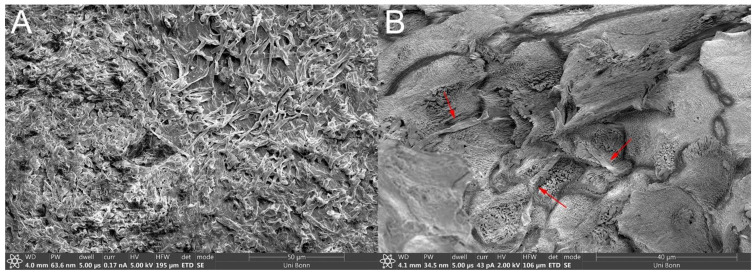
Scanning electron microscopy images of infected 400-µm bovine hoof sheets: (**A**) dense growth of fungi over the entirety of the membrane surface, and a (**B**) cross-section fracture reveals fungal growth inside the membrane matrix, fungal hyphae, indicated by arrows.

µCT and SEM images depicted a smooth-to-wavy surface texture with a multitude of porous structures on the membrane’s surface (Figure 1A,B). Some structures on the surface appeared to be during in SEM, while some were visibly open, with a diameter of up to roughly 50 µm. Further examination by µCT revealed pores extending coherently throughout the whole membrane transversally as brighter areas against the darker keratin matrix (Figure 2B,C). Exemplary focusing of such a pore in the middle of the membrane’s diameter (yellow cross) showed a hollow area visible from each axis that continued transversally as a dark crack. Further FIB milling of the matrix around these pores and the subsequent imaging of the resulting cross-section revealed that, although pores may seem to be closed or superficial, hollow structures continued beneath the surface, connecting to deeper membrane layers (Figure 1B). SEM imaging of the infected BHS showed the dense, superficial growth of fungal hyphae, completely covering the membrane’s surface (Figure 3A). Imaging of a fracture cross-section revealed that fungi also grew into the membrane (Figure 3B).

### 3.2. Solid State of PAT and LAC

A solid-state analysis of PAT and LAC showed an absence of crystallinity for 1% and 10% BFZ formulations, whereas, for the 20% drug load, crystals could be observed with the PLM and BFZ melting peaks and reflexes that occurred in the thermogram and diffractogram. For the PLM photographs, thermograms, and diffractograms, please refer to the Supplementary Materials (Figures S1–S3).

### 3.3. Permeation across Synthetic Membrane

**Figure 4:**
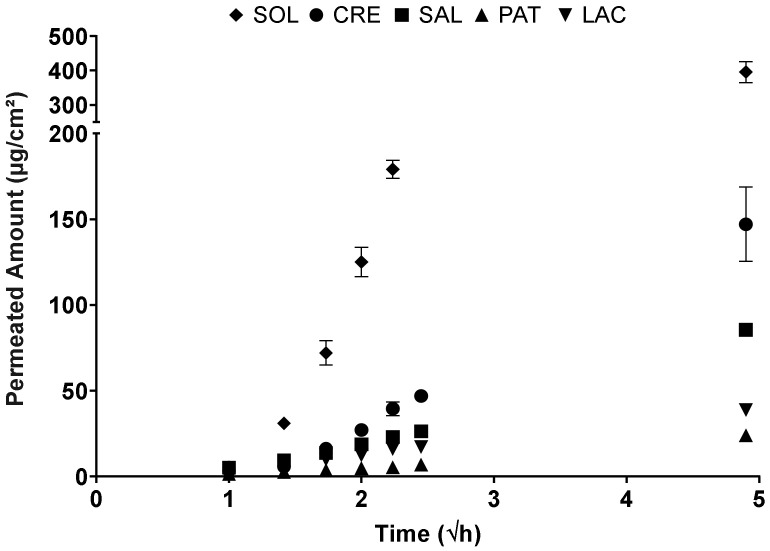
Permeation of bifonazole (BFZ) out of the solution (SOL), Canesten^®^ Extra Creme (CRE), Canesten^®^ Extra Salbe (SAL), patch (PAT), and lacquer (LAC) across a synthetic regenerated cellulose membrane (mean ± SD).

Plotting the permeated amount of BFZ per diffusion area against the square root of time allowed an assessment of the flux for all the dosage forms (Figure 4). SOL’s permeation, followed a sigmoidal pattern, resulted in the highest total permeation after 24 h. While the instant release dosage forms CRE and SAL showed similar releases over the first 3 h, CRE released roughly 1.7 times the amount of BFZ compared to SAL after 24 h. In comparison, the extended-release dosage forms released significantly less than BFZ. LAC released about 26% of semisolid reference CRE, whereas the amount of BFZ liberated from PAT amounted to 16% of CRE.

### 3.4. Permeation across Bovine Hoof Sheets 

**Figure 5:**
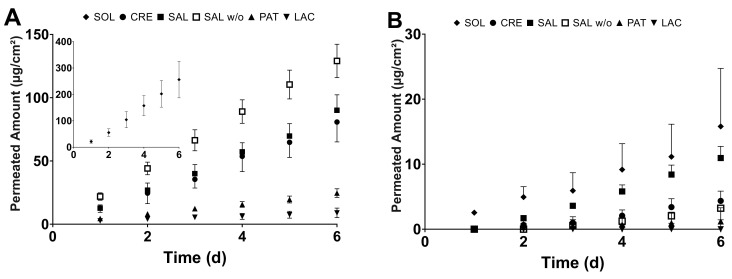
Permeation of bifonazole (BFZ) out of the solution (SOL), Canesten^®^ Extra Creme (CRE), Canesten^®^ Extra Salbe (SAL), Canesten^®^ Extra Salbe without urea (SAL *w*/*o*), patch (PAT) and lacquer (LAC) across (**A**) 100-µm and (**B**) 400-µm healthy bovine hoof sheets (mean ± SD).

Linear permeation profiles for all the dosage forms over the course of 6 days were established for membranes 100 µm and 400 µm thick (Figure 5). For CRE and SAL, the permeations were linear over the course of 14 d at least (data not shown). For 100-µm-thick membranes, SOL clearly showed the highest permeation, with 256.0 ± 69.1 µg/cm^2^ of permeated BFZ after 6 d. The immediate release dosage forms CRE and SAL showed similar permeation profiles with no statistically significant differences between them, resulting in 80.8 ± 15.7-µg/cm^2^ and 90.0 ± 12.3-µg/cm^2^ permeating. Permeated amounts from the PAT and LAC were distinctly lower than with the other formulations, with 24.6 ± 3.4 µg/cm^2^ permeated out of PAT in contrast to LAC with only 9.0 ± 3.8 µg/cm^2^.

For 400-µm-thick BHS, SOL still showed the highest onset of permeation and resulted in 15.8 ± 8.9 µg/cm^2^ permeated after 6 d. However, while showing a slower onset, the SAL was similar over the course of 6 d, releasing 11.0 ± 1.8 µg/cm^2^ into the receiver medium. While no difference could be observed in the 100-µm setting, the SAL clearly delivered more BFZ than CRE, which only showed a permeation of 4.4 ± 1.5 µg/cm^2^, similar to PAT with 1.2 ± 2.5 µg/cm^2^. In the case of LAC, no permeation could be observed in any FDC over the course of the experiment.

Regarding the permeation of SAL *w*/*o* after 6 d, 129.1 ± 13.2 µg/cm^2^ permeated across 100-µm BHS, whereas 3.2 ± 1.8 µg/cm^2^ permeated in the case of 400 µm. 

**Figure 6:**
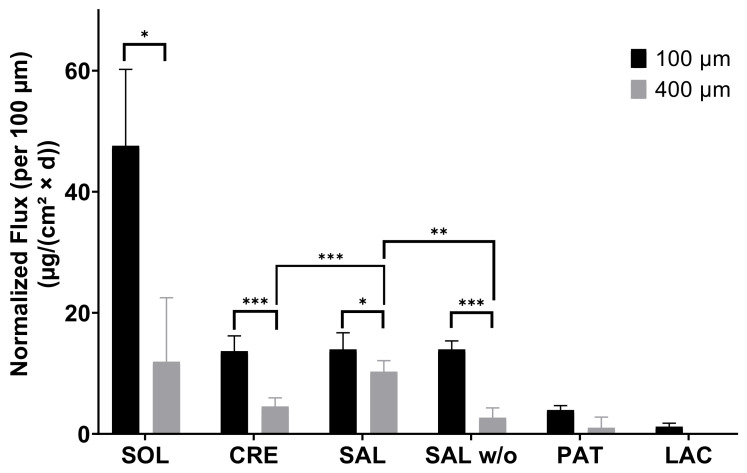
Respective fluxes of bifonazole through 100-µm and 400-µm healthy bovine hoof sheets (mean ± SD). Significance levels: * *p* < 0.05; ** *p* < 0.01; *** *p* < 0.001.

In general, increasing the membrane’s thickness to 400 µm led to a disproportional decrease in the flux when compared to 100 µm for all dosage forms, as shown by normalization to a membrane thickness of 100 µm (Figure 6). The normalized flux was significantly different for every immediate release dosage form. Between 100 and 400 µm, normalized fluxes for SOL, CRE, SAL, SAL *w*/*o* and PAT differed by factors of 4.0, 3.0, 1.4, 5.2 and 4.0, respectively, while, for LAC, no factor could be derived due to no observable permeation across 400 µm. Furthermore, no significant difference could be observed between CRE, SAL, and SAL *w*/*o* in the 100-µm setting. However, increasing the membrane thickness to 400 µm resulted in a significantly different flux between the CRE and SAL, as well as an increased permeation of SAL compared to SAL *w*/*o*.

### 3.5. Permeation across Infected Bovine Hoof Sheets

**Figure 7:**
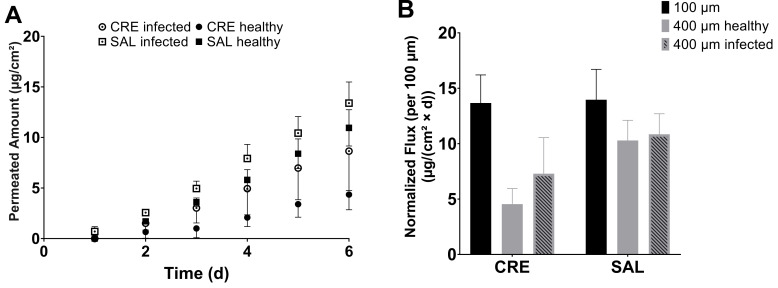
(**A**) Permeation of bifonazole from Canesten^®^ Extra Creme (CRE) and Canesten^®^ Extra Salbe (SAL) across 400-µm bovine hoof sheets and healthy and infected with *T. rubrum* (mean ± SD). (**B**) Respective fluxes of bifonazole through 100-µm and 400-µm healthy and infected membranes (mean ± SD).

Infecting 400-µm-thick BHS with *T. rubrum* for 9 d provided membranes with nonincreased thicknesses to healthy BHS, which were of sufficient robustness for use in FDC experiments. The surfaces’ colors changed from opaque white to a reddish brown in the infected state. The permeation profiles for CRE and SAL exhibited a similar flux to healthy 400-µm membranes that could be observed for SAL, resulting in a 13.4 ± 2.1-µg/cm^2^ amount permeated (Figure 7). Then again, the flux of CRE increased in the infected model over the noninfected one and approached that of SAL, with 8.7 ± 3.9 µg/cm^2^ permeated after 6 d. In addition to the permeation results, the infected BHS surface lost its discoloring when treated with SAL, undergoing careful daily formulation exchanges with a plastic spatula. Comparably, the BHS surface of CRE-treated BHS whose donor was exchanged with cotton swabs did not alter their color in a similar fashion (see Supplementary Materials Figure S4).

## 4. Discussion

When implementing BHS as a surrogate model for human nails, Mertin and Lippold found BHS to be less dense, with an increased swelling capacity compared to human nails, resulting in a higher permeability [15]. However, upon verifying the integrity of their membranes via SEM imaging, they stated an absence of pores but existence of cavities [13]. In contrast to their findings, using the FIB milling technique combined with SEM imaging, these cavities were shown not to be superficial but connect to deeper membrane levels. The additional investigation of these structures with µCT imaging further confirmed the existence of connected, transversally proceeding hollow cracks. The porosity of BHS and human nails was investigated by Nogueiras-Nieto et al. using mercury intrusion porosimetry [16]. Agreeing with our findings, their study showed that dry BHS had a pronounced porosity at the surface; yet, the internal structure of BHS had a low porosity. The hydration of both human nails and BHS increased the porosity and interconnection of the keratinous network. As revealed by our imaging, the decline of porosity in the deeper membrane levels in these studies might be due to a blockade of porous tunnels by keratinous debris. The properties of bovine claw horn were investigated by Baillie et al. [17]. Using a light microscope, they demonstrated continuous tubules running through the bovine hoof, which were shown to be hollow in SEM fracture specimens by Wang et al. [18]. Despite the hydration, freezing, and cutting steps in preparation of the BHS, these tubules were not closed, as revealed by µCT and FIB-SEM. Thus, it is very likely that channels filled with the receiver medium are present in FDC experiments. These channels presumably act as shunt routes, facilitating permeations. Therefore, an increased membrane thickness might reduce the effect of such routes through the increased chance of debris in tubules and a longer route of diffusion for APIs and subsequently lead to more appropriate datasets than with thinner BHS.

Bifonazole transport across a regenerated cellulose membrane showed a distinct order for the different dosage forms. SOL, CRE, and SAL, in that order, showed much more pronounced permeations compared to the matrix forming LAC and PAT, which could be attributed to the presumed increased diffusivity of BFZ in these liquid and semisolid dosage forms. The permeated amount out of LAC was higher than PAT, despite a presumably lower diffusivity of BFZ, which may be due to a lower hydrophobicity of RLPO compared to DT, enabling a more efficient solvent uptake and diffusion through the matrix. As the dialysis membrane does not possess the retention potential for small molecules, these findings can be interpreted as a BHS membrane with an infinite number of pores and short ways of diffusion. Similar to the synthetic membrane, the 100-µm-thick BHS allowed for differentiation between liquid, semisolid, and solid formulations with the same drug load of BFZ. However, while SOL maintained the highest permeation and the rankings of LAC and PAT reversed, possibly due to less direct contact of the dosage form with the receiver medium, the former difference between CRE and SAL became insignificant, as the barrier properties of BHS became the limiting factor of diffusion in contrast to the dialysis membrane. Naumann et al. were able to assess the permeation of a novel antifungal agent from a solution, a lacquer, a hydrogel, and a colloidal carrier system [19]. In their study, using an FTIR-ATR diffusion cell, they showed the highest permeation from the solution followed by emulsion, with the lacquer and hydrogel being significantly worse, thus confirming the revealed applicability of 60–150-µm-thick BHS for the general comparison of dosage forms. Further studies were conducted by Monti et al., who successfully compared the permeations of amorolfine and ciclopirox from the same hydrophilic vehicle using 80–120-µm BHS [20]. Their report reinforced these membranes as tools that allow quantifiable and comparable permeation results from lacquers for different APIs.

The effects of penetration enhancers have been reported for BHS of 60-µm and 200-µm thickness for lacquers [12,21]. In these studies, urea was shown to act as a potent enhancer of transungual permeation, facilitating the delivery of terbinafine hydrochloride and amorolfine hydrochloride. However, urea seemingly did not enhance the permeation from SAL compared with CRE, and the permeation from SAL *w*/*o* was even higher in the 100-µm BHS system. Regardless, the flux was not significantly different between SAL, SAL *w*/*o*, and CRE, whereas, across 400-µm-thick membranes, the BFZ permeation of SAL was significantly higher than that of CRE or SAL *w*/*o*. Therefore, although already much more limiting than the dialysis membrane, in the 100-µm BHS setup, the high amount of BFZ intrinsically released from immediate-release dosage forms overshadowed the penetration-enhancing effects when confronted with too weak of a barrier. In addition, effects of lower viscosity might have resulted in a faster onset of permeation for less viscous SAL *w*/*o* in contrast to pasty SAL, explaining the resulting difference in the permeated amount across 100-µm BHS. Using 200–250-µm-thick BHS, Park et al. investigated the influence of lipophilic and hydrophilic permeation enhancers on the transungual delivery of hydroalcoholic and oily eficonazole solutions [22]. For hydrophilic enhancers, their study allowed accurate discrimination between the respective formulations. Further studies with penetration enhancers were performed by Cutrín-Gómez et al., in which they used 300–700-µm membranes and studied the enhancers’ effects on the membrane surface morphology and porosity [23]. They concluded that enhancers, such as sodium laureth sulfate, or acetylcysteine had a porosity-increasing effect on the matrix. Thus, thicker membranes, which provide more keratinous material in comparison to the already existing porous structures, would yield an increased response. Since the permeations from immediate release dosage forms throughout increased disproportionally to the thickness of the respective membrane, independent of any penetration-enhancing substances, the enhancing effects of the formulations could be neglected, and the pores in the membrane were identified as a likely cause by SEM and µCT imaging. The choice of the right membrane in this particular test system is of utmost importance to obtain the most reliable results for the use of a formulation. Therefore, to simulate infection of the nail, BHS of 400-µm thickness were infected with *T. rubrum.* The commercial products CRE and SAL were chosen for further investigation in this arrangement to best mimic actual use conditions, where the application of SAL is recommended for two weeks prior to application of CRE to evaluate whether the infected model would maintain the observed differences in the healthy setup, thus supporting this recommendation. Although SAL showed similar permeations to healthy membranes, the permeations from CRE were increased in the infected model. As shown by the SEM images, the fungi grew into the membrane rather than just superficially. Hence, an increase in porosity due to digestion of the keratinous matrix by fungi is highly probable. This higher porosity of the membrane increased the flux mainly for CRE, which benefited more from the increased porosity analogous to the 100-µm BHS, correlating with higher intrinsic liberation characteristics. Although the delivery from SAL was not increased, the infected matrix was removed with a daily exchange of the donor, as the keratolytic effect of urea probably broke up the outermost layers, facilitating removal in contrast to CRE. An increase in porosity by fungal infection for human nails and resulting trends for increased flux for ciclopirox olamine and clobetasol propionate were reported by Cutrín-Gómez et al. [24]. BHS and keratin films of human hair were infected with *T. rubrum* by Lusiana et al. and Kracht et al. [14,25]. In their work, the infected membranes were inoculated on agar plates, an antifungal formulation was applied, and the fungal growth was assessed after a period of time to determine the efficacy of the formulations. While this setup allows for the rapid screening of multiple formulations, it does not allow for the assessment of permeation parameters, unlike the use of infected BHS in transungual FDC experiments. In addition, the infected FDC setup enabled the discovery of diverse formulation effects on the nail membrane, such as keratolysis, as demonstrated by removal of the superficially infected matrix by treatment with SAL, which could not be elucidated in previous studies.

In this work, BFZ was studied as a model drug, being a representative candidate in this therapeutic indication, with the main focus on establishing an ameliorated tool for comparative studies between the different dosage forms. The observations underline the importance of selecting an appropriate membrane when planning studies of permeations from ungual formulations. Thus, future studies with different APIs and dosage forms could benefit from the demonstrated applicability of different BHS thicknesses and infection statuses. Although seemingly different permeation mechanisms were involved with the respective dosage forms in the dependance of membrane thickness and infection, the observed differences could not completely be explained mechanistically yet. However, this modified model offers a foundation for a more rational experimental setup in formulation development. Nevertheless, exploring further mechanistical insights into the influence of thickness and infection will be helpful to refine the model’s applicability to other therapeutic scenarios.

## 5. Conclusions

Bovine hoof sheets have rightfully been established as a surrogate for human nails in in vitro test systems, as they allow for rather physiological, yet still sufficiently fast, assessments of permeation characteristics of typically employed formulations, most of all lacquers. However, for liquid or semisolid formulations, shunt routes, in the form of naturally grown pores, drastically increase the permeation upon lowering the membrane’s thickness. Only with membranes thick enough to effectively limit diffusion does it become possible to reach a reasonable resolution between SAL and CRE in order to detect the potential permeation-enhancing effects, as found in this study, with urea in SAL. The infection of BHS increased the permeation similarly to lowering the membrane thickness and further allowed for an inspection of the formulations’ effects on removal of the infected matrix. This model design significantly increased the nail thickness, enabling to identify and prove a therapeutic regimen essentially based on the dosage form design, such as in the urea containing semisolid formulation given here combining a rapid drug delivery into the nail matrix with simultaneous removal of the nail matrix. Accordingly, standards would be highly useful in guiding the selection of thickness and infection status of bovine hoof sheets in view of the particular dosage form and the therapeutic regimen.

## Data Availability

Not applicable.

## Supplementary Materials

The following are available online at https://www.mdpi.com/article/10.3390/pharmaceutics13122098/s1: Figure S1: PLM images of PAT and LAC. Figure S2: X-ray diffractograms of PAT and LAC. Figure S3: Thermograms of PAT and LAC. Figure S4: Surfaces of infected 400-µm BHS treated with SAL and CRE.
