# Supplementary material for: Improving Transungual Permeation Study Design by Increased Bovine Hoof Membrane Thickness and Subsequent Infection
Source: Pharmaceutics. 2021 Dec 6;13(12):2098. doi: 10.3390/pharmaceutics13122098 (PMC8707648; doi:10.3390/pharmaceutics13122098)
Supplement: Supplementary file 1 [file pharmaceutics-13-02098-s001.zip › pharmaceutics-1462556-supplementary.pdf]

Supplementary Materials

Improving Transungual Permeation Study Design by Increased Bovine Hoof Membrane Thickness and Subsequent Infection

Sebastian Kappes [1], Thilo Faber [1], Lotta Nelleßen [1], Tanju Yesilkaya [2], Udo Bock [3] and Alf Lamprecht [1,4,*]

[1] Department of Pharmaceutics, Institute of Pharmacy, University of Bonn, Gerhard-Domagk-Str. 3, 53121 Bonn, Germany; kappes.sebastian@uni-bonn.de (S.K.); thilo.faber@uni-bonn.de (T.F.); s6annell@uni-bonn.de (L.N.)

[2] Bayer Vital GmbH, 51373 Leverkusen, Germany; tanju.yesilkaya@bayer.com

[3] Bock Project Management, 54456 Tawern, Germany; udo.bock@bock-pm.com

[4] PEPITE EA4267, University of Burgundy/Franche-Comté, 25000 Besançon, France

* Correspondence: alf.lamprecht@uni-bonn.de; Tel.: +49-228-735-243; Fax: +49-228-735-268

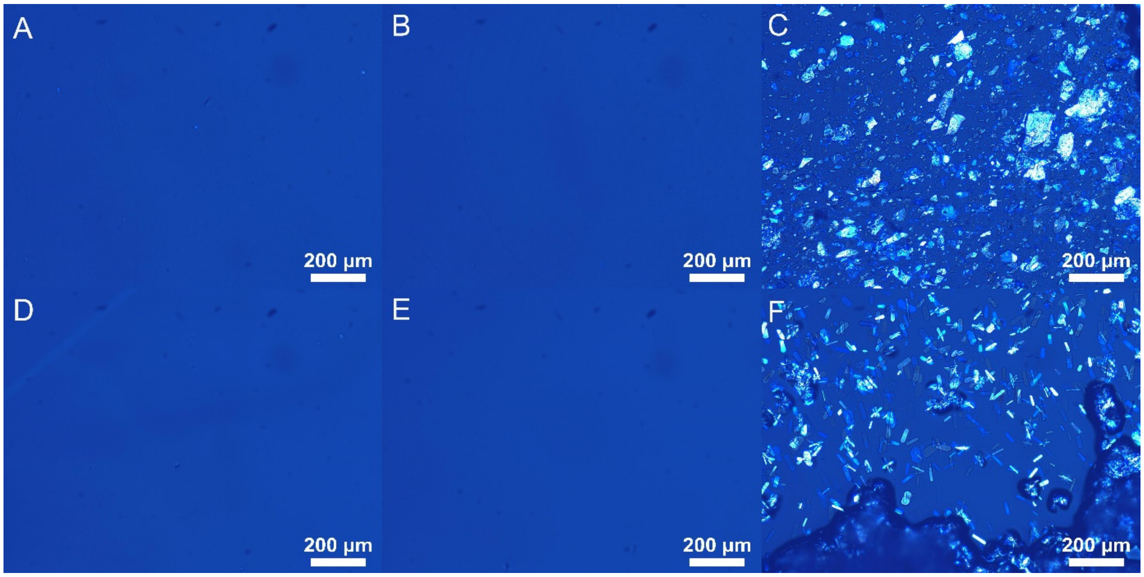

Figure S1. Polarized light microscopy images of a (**A**) patch (PAT) placebo, (**B**) 10% PAT, (**C**) 20% PAT, (**D**) lacquer (LAC) placebo, (**E**) 10% LAC and (**F**) 20% LAC.

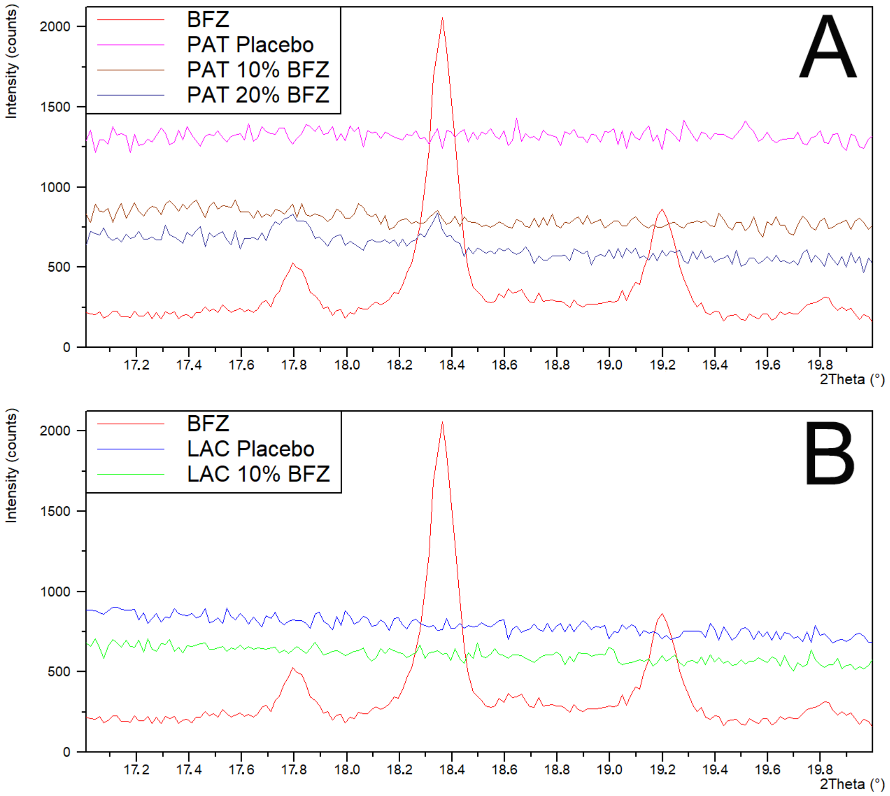

Figure S2. X-ray diffractograms of a (**A**) patch (PAT) and (**B**) lacquer (LAC) containing bifonazole (BFZ).

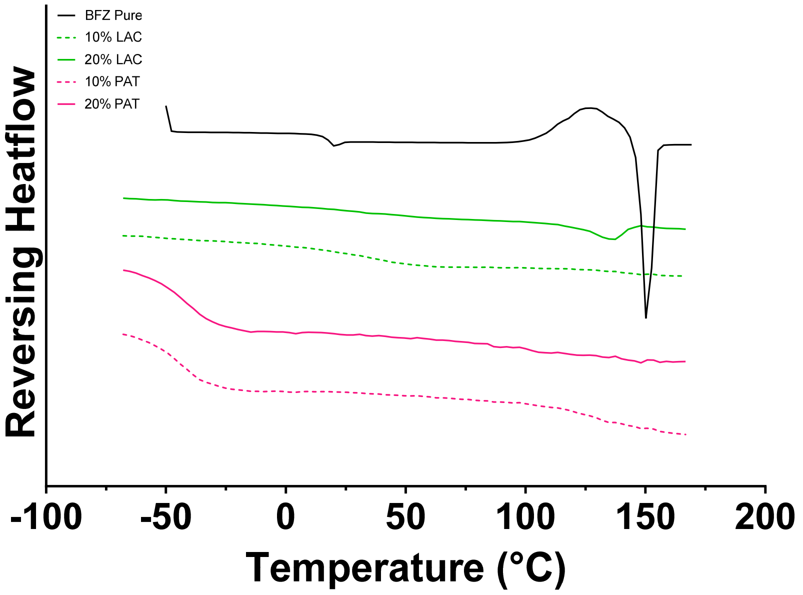

Figure S3. Thermograms of pure bifonazole (BFZ), 10 and 20% patch (PAT), and lacquer (LAC).

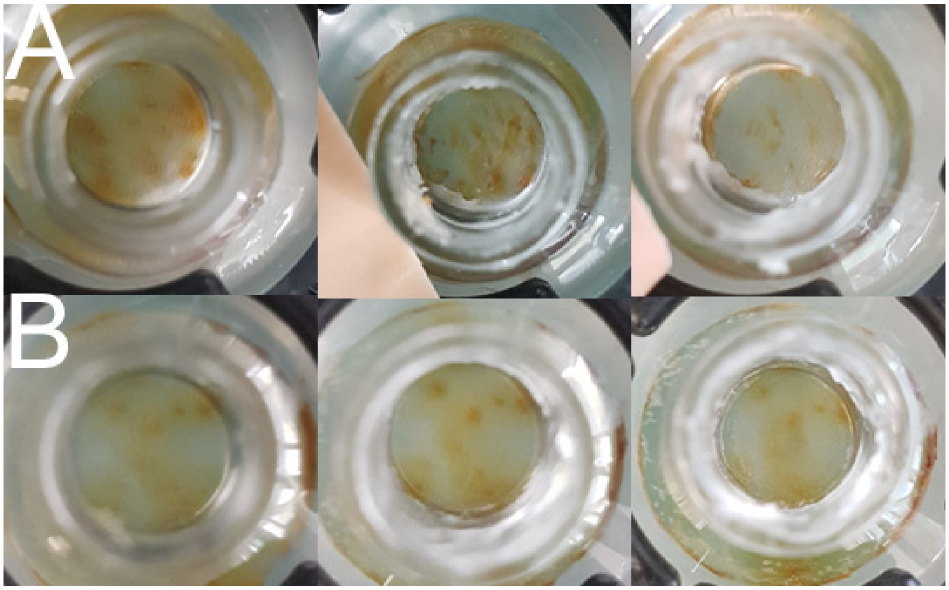

Figure S4. Surfaces of infected 400-μm bovine hoof sheets treated with (**A**) Canesten® Extra Salbe and (**B**) Canesten® Extra Creme at 0 d, 3 d and 6 d from left to right.
